# P-1853. Peripheral blood monocytes pro-inflammatory and anti-inflammatory cytokine release in elderly patients with heart failure with reduced ejection fraction

**DOI:** 10.1093/ofid/ofae631.2014

**Published:** 2025-01-29

**Authors:** Yasuiko Mitsuke, Kazuhiro Itoh, Hiromichi Iwasaki

**Affiliations:** NHO Awara National Hospital, Awara, Fukui, Japan; NHO Awara National Hospital, Awara, Fukui, Japan; University of Fukui Hospital, fukui, Fukui, Japan

## Abstract

**Background:**

Heart failure with reduced ejection fraction (HFrEF) has benn reported to characterize by sub-clinical inflammation status. There are few reports about changes in selected biomarkers of inflammation concomitant with the release of pro-inflammatory and anti-inflammatory cytokines by monocyte in elderly patients with HFrEF.

**Methods:**

We measured plasma levels of the cytokines in 62 elderly Japanese HFrEF patients (aged 85.8 ± 2.7 years, left ventricular ejection fraction 33.7 ± 3.5%) and 20 healthy controls. Peripheral blood monocytes (PBM) obtained from HFrEF patients were stimulated by LPS (10 μg/ml) in the absence or the presence of adenosine (10 μmol/l). After 6hr incubation, the cytokines levels in the culture supernatants were measured by ELISA.

**Results:**

Plasma levels of TNF-alpha, IL-6, IL-10, and IL-12p40 were significantly greater in HFrEF patients than in controls. Plasma IL-12p40 levels of HFrEF patients showed significant correlations with IL-6 and IL-10. In vitro study, adenosine inhibited the generation of pro-inflammatory cytokines from LPS-stimulated PBM (TNF-alpha: 764.2±32.3 to 242.5±55.3pg/ml, IL-6: 82.3±13.5 to 21.4±4.2 pg/ml, IL-12p40: 164.8±23.5 to 18.2±5.1, all p< 0.01), while it augmented IL-10 generation (29.2±6.1 to 54.9±7.8pg/ml, p< 0.01) significantly.

**Conclusion:**

Our data indicated plasma levels of pro-inflammatory and anti-inflammatory cytokine were elevated in eldely patients with HFrEF relating. Adenosine may represent a new pharmacological intervention in such HFrEF patients,the clinical significance of these findings deserves further investigation

**Disclosures:**

**All Authors**: No reported disclosures

